# Delta radiomics model for the prediction of progression-free survival time in advanced non-small-cell lung cancer patients after immunotherapy

**DOI:** 10.3389/fonc.2022.990608

**Published:** 2022-10-06

**Authors:** Dong Xie, Fangyi Xu, Wenchao Zhu, Cailing Pu, Shaoyu Huang, Kaihua Lou, Yan Wu, Dingpin Huang, Cong He, Hongjie Hu

**Affiliations:** ^1^ Department of Radiology, Sir Run Run Shaw Hospital, Zhejiang University School of Medicine, Hangzhou, China; ^2^ Department of Radiology, Shaoxing Second Hospital, Shaoxing, China; ^3^ Department of Radiology, Ningbo Medical Center LiHuili Hospital, Ningbo, China

**Keywords:** non-small-cell lung cancer, radiomics, delta, immunity, prediction model

## Abstract

**Objective:**

To assess the validity of pre- and posttreatment computed tomography (CT)-based radiomics signatures and delta radiomics signatures for predicting progression-free survival (PFS) in stage III-IV non-small-cell lung cancer (NSCLC) patients after immune checkpoint inhibitor (ICI) therapy.

**Methods:**

Quantitative image features of the largest primary lung tumours were extracted on CT-enhanced imaging at baseline (time point 0, TP0) and after the 2^nd^-3^rd^ immunotherapy cycles (time point 1, TP1). The critical features were selected to construct TP0, TP1 and delta radiomics signatures for the risk stratification of patient survival after ICI treatment. In addition, a prediction model integrating the clinicopathologic risk characteristics and phenotypic signature was developed for the prediction of PFS.

**Results:**

The C-index of TP0, TP1 and delta radiomics models in the training and validation cohort were 0.64, 0.75, 0.80, and 0.61, 0.68, 0.78, respectively. The delta radiomics score exhibited good accuracy for distinguishing patients with slow and rapid progression to ICI treatment. The predictive accuracy of the combined prediction model was higher than that of the clinical prediction model in both training and validation sets (P<0.05), with a C-index of 0.83 and 0.70, respectively. Additionally, the delta radiomics model (C-index of 0.86) had a higher predictive accuracy compared to PD-L1 expression (C-index of 0.50) (P<0.0001).

**Conclusions:**

The combined prediction model including clinicopathologic characteristics (tumour anatomical classification and brain metastasis) and the delta radiomics signature could achieve the individualized prediction of PFS in ICIs-treated NSCLC patients.

## Introduction

Non-small-cell lung cancer (NSCLC) is one of the most prevalent malignancies with regards to incidence and mortality, which accounts for 85% of all cases. Despite recent advances in lung cancer treatment, the five-year survival rate of NSCLC patients is still low, at 15% (1). During the last decade, the recognition of the critical role of immune system evasion in cancer pathogenesis has stimulated the development of immune checkpoint inhibitors (ICIs) for treating various malignancies and also changed the treatment outlook for patients with advanced NSCLC without any targeted mutations. Compared to chemotherapy, immune checkpoint blockade against programmed death-1 (PD-1) or programmed death-ligand 1 (PD-L1) has prolonged the overall survival (OS) of advanced NSCLC patients (2–6). Despite the successful application of immunotherapy, only 15-30% patients showed improved OS and/or progression-free survival (PFS) according to previous studies (7, 8).

Although PD-L1 expression in tumour cells is widely used as a biomarker to select patients for immunotherapy (6, 9, 10), the association between PD-L1 expression and treatment efficacy of ICIs remains inexact. It has been shown that atezolizumab treatment improves survival in NSCLC independent of PD-L1 expression status (11, 12), and the results of several other studies have shown that cases with PD-L1-negative tumours still obtain a significant benefit from ICI treatment (3, 4, 10). In addition, the heterogeneity of PD-L1 protein expression, nonstandardized assays, instability of tissue specimens, PD-L1 copy number status and other factors limit PD-L1 expression as a predictive biomarker (13–15). Therefore, the inadequacy of current biomarkers urgently requires the discovery of novel predictive biomarkers for selecting patients who can benefit from ICI therapy.

Radiomics is a noninvasive diagnostic tool that extracts quantitative imaging features from traditional clinical images for diagnosis and can generate imaging biomarkers to provide guidance for clinical decision making (16). A computed tomography (CT) image-based radiomics method has been used to establish a prediction model for the differential diagnosis (17, 18), clinical staging (19–21), evaluation of treatment efficacy (22, 23), and gene mutation prediction (24–27) of lung cancer. Although clinical staging remains the main method for the prediction of survival time in NSCLC patients, there are large differences in treatment response and prognosis among patients with the same staging, showing that prognostic stratification is crucial for individualized treatment. Several authors have assessed the relationship between radiomics and the OS of NSCLC patients and developed survival prediction models for lung cancer patients based on radiomics approaches (28–33). The treatment-induced changes in radiomics features (RFs) can be captured by delta RFs, allowing us to describe their longitudinal changes, and are more suitable for monitoring the therapeutic response (34, 35).

We designed a retrospective study to explore the potential application of a CT image-based radiomics model (RM) for predicting the probability of progression on individualized ICI treatment. To deeply examine advanced NSCLC patients receiving immunotherapy, we isolated thousands of pre- and posttherapy CT features and built pretherapy, posttherapy and delta RMs. The optimal RM was selected for the risk stratification of PFS in advanced NSCLC patients. Finally, we combined the radiomics signature and clinicopathological features to build a new prediction model to provide reliable individualized clinical recommendations for the probabilities of PFS at 7 months and 1 year of treatment with ICIs.

## Materials and methods

### Patients

This was a retrospective analysis of 97 NSCLC patients treated with ICIs in Run Run Shaw Hospital, Zhejiang University School of Medicine, from January 2016 to November 2021, including 88 males and 9 females. Tumour staging was conducted in accordance with the eighth edition of the American Joint Committee on Cancer TNM staging criteria (36), and all patients had pathologically confirmed stage III-IV NSCLC. Clinicopathological data, laboratory tests, and imaging data were acquired from the patients, such as sex, age, smoking history, tumour anatomical classification, pathological type, tumour markers, TNM staging, line of treatment, and treatment strategy. The patients were assigned randomly to a training cohort of 68 patients and a validation cohort of 29 patients according to a ratio of 7:3. The training cohort and validation cohort were employed to construct and verify the prediction model, respectively. The study was approved by the hospital ethics committee (Grant No.: Research 20220222-33), and was carried out in compliance with the Declaration of Helsinki.

The following inclusion criteria were used: (1) NSCLC with histologic confirmation; (2) ICI therapy in a first- or later-line setting; and (3) complete baseline demographics prior to treatment. The exclusion criteria were as follows: (1) baseline imaging or follow-up after 2-3 cycles of treatment without contrast-enhanced CT; (2) the lesion boundaries could not be accurately evaluated in contrast-enhanced CT images; and (3) the interval between baseline imaging and the initial immunotherapy exceeded 4 weeks.

### CT image acquisition and interpretation

The pretreatment and follow-up CT scans were obtained by 64-slice LightSpeed VCT (**Supplementary Data**). Two radiologists (reader A and B, with 13 and 7 year’s experience in chest CT interpretations, respectively) performed independent evaluations. The observation indexes included the selection of the target lesion, anatomical classification of the tumour, tumour boundary, TNM stage, etc.

In this study, treatment efficacy was evaluated by detecting PFS, defined as the time from the initiation of ICI therapy to the confirmation of disease progression or disease-related death. To assess whether the target lesion had progressed, the Response Evaluation Criteria in Solid Tumours (RECIST, v1.1) (37) was used.

### Tumour segmentation

**Figure 1:**
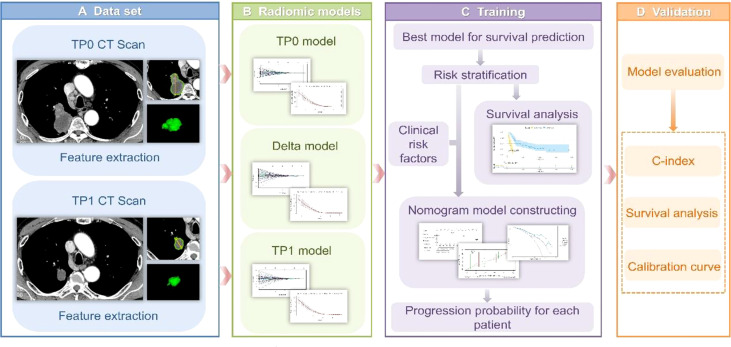
**(A)** Tumour segmentation. The figure shows baseline CT images of target lesions and CT images after 2-3 cycles of treatment, tumour contours and three-dimensional visualizations. **(B)** TP0, TP1 and delta RMs were established based on pretherapy, posttherapy and delta RFs. **(C)** The best RM was selected for the risk stratification of drug resistance, while a nomogram was established for individualized prognosis prediction. **(D)** All prediction models were validated in the validation cohort.

Reader A segmented the target lesions of all the patients layer-by-layer on enhanced imaging at baseline (time point 0, TP0) and after the 2nd-3rd immunotherapy cycles (time point 1, TP1). The ITK-SNAP software (v3.6.0, www.itksnap.org) was used to conduct three-dimensional (3D) manual segmentation. Examples are shown in **Figure 1A**. To ensure accuracy and reproducibility, 10 patients were randomly chosen one month after the first segmentation, and readers A and B segmented the region of interest (ROI) of the lesions at TP0 and TP1 and extracted features. The inter- and intraclass correlation coefficients (ICCs) were employed to assess the consistency of features, and the ICC of >0.75 was regarded as a marker of good reliability.

### Radiomics feature extraction and data normalization

The PyRadiomics package was applied to analyse the segmentation data and isolate phenotypic features from the tumour regions after manual segmentation. To standardize all voxel sizes among patients, the CT images were resampled to 2-mm resolution in all 3 directions. RFs were extracted from each 3D ROI. Delta RFs were regarded as the net changes in RFs between TP0 and TP1. Delta RFs = Feature (TP1)- Feature (TP0). In addition, all features were z score normalized using Excel.

### Feature screening and signature construction

In the training cohort, the most significant predictive features were selected by the least absolute shrinkage and selection operator (LASSO)-penalized Cox proportional hazards regression model, and finally, a radiomics signature containing critical features and their corresponding weight coefficients was constructed. The radiomics score (Rad-score) was estimated for all patients, and was utilized to construct the RM. The optimal cut-off value of the Rad-score was assessed according to X-tile software v3.6.1, and the patients were assigned to Rad-score high and Rad-score low subgroups, representing the rapid-progression (RP) and slow-progression (SP) subgroups treated with ICIs, respectively. The relationships between the RFs and PFS were determined by Kaplan–Meier survival analysis in training and validation cohorts, and the log-rank test was applied to examine whether there was statistical difference in survival between the RP and SP subgroups.

### Construction of predictive models

The TP0 RM, TP1 RM and delta RM were constructed to determine the predictive accuracy for the efficacy of immunotherapy in advanced lung cancer (**Figure 1B**). We also constructed a clinical prediction model using significant clinicopathological characteristics *via* univariate and multivariate Cox regression analyses. To explore whether the clinical characteristics combined with the RFs can further enhance the model performance, a combined model was established. The concordance index (C-index) was applied to evaluate the model performance. The calibration curve was applied to determine the agreement between the predicted PFS of the prediction model and the actual observed PFS. Decision curve analysis (DCA) was conducted to assess the applicability of the prediction models by calculating the net benefits at various threshold probabilities. See **Figures 1C, D**.

### Statistical methods

Statistical tests were performed with SPSS v26.0 and R (www.r-project.org, version 4.1.2). The Mann–Whitney test was employed for continuous variables, which were expressed as the median (interquartile range) [M (Q1, Q3)]. The Fisher’s exact test or chi-square test was applied for categorical variables, which were expressed as percentages [n (%)]. A multivariate Cox regression analysis with the backwards elimination method was used to develop the best model integrating clinical factors and RFs. P-values of <0.05 were deemed as significant differences.

## Results

### Treatment, clinical characteristicsand PFS

The demographic and clinicopathological characteristics of the 97 patients are indicated in **Table 1**. Fifty-three (54.6%) of all patients received PD-1 ICIs (camrelizumab, sintilimab, tislelizumab or nivolumab) or PD-L1 ICIs (atezolizumab) monotherapy. The remaining 44 (45.4%) patients were treated with the combination of immunotherapies, ICIs in combination with chemotherapeutic agents (gemcitabine + cisplatin, paclitaxel + carboplatin) and/or antiangiogenic agents (mainly bevacizumab, Endo, anlotinib, and afatinib). The median time between TP0 and the initiation of immunotherapy was 10 (5, 16) days; the median time between TP0 and TP1 was 59 (51, 68) days; and the follow-up periods for training and validation cohorts were from January 19, 2016, to November 12, 2021, and from January 21, 2016, to November 15, 2021, respectively. A total of 82 (84.5%) patients showed PD, and 15 (15.5%) patients were lost to follow-up. During the follow-up, the median PFS of the training cohort was 6.9 (4.3, 19.7) months, and that of the validation cohort was 7.3 (2.8, 14.7) months; but no significant difference was found between two cohorts. Furthermore, the differences in demographic and clinicopathological characteristics between the training and validation cohorts were not statistically significant (P>0.05) (**Supplementary Table S1**).

**Table 1:** Baseline data and PFS of the training and validation cohorts.

Demographic or clinicopathologic characteristic, PFS		Training cohort (N=68)			Validation cohort (N=29)	
	No.	Rapid (N=20)	Slow (N=48)	No.	Rapid (N=13)	Slow (N=16)
Sex (%)						
Male	62	17 (27)	45 (73)	26	11 (42)	15 (58)
Female	6	3 (50)	3 (50)	3	2 (67)	1 (33)
Age, years (%)						
≤65	25	7 (28)	18 (72)	16	6 (38)	10 (62)
>65	43	13 (30)	30 (70)	13	7 (54)	6 (46)
Smoking history (%)						
Smoker	35	13 (37)	22 (63)	15	8 (53)	7 (47)
Non-smoker	33	7 (21)	26 (79)	14	5 (36)	9 (64)
Anatomical classification (%)						
Central type	36	15 (42)	21 (58)	17	8 (47)	9 (53)
Peripheral type	32	5 (16)	27 (84)	12	5 (42)	7 (58)
Pathological type (%)						
Squamous cell	45	15 (33)	30 (67)	23	10 (43)	13 (57)
Adenocarcinoma	23	5 (22)	18 (78)	6	3 (50)	3 (50)
Lung metastasis (%)						
Yes	20	9 (45)	11 (55)	13	6 (46)	7 (54)
No	48	11 (23)	37 (77)	16	7 (44)	9 (56)
Brain metastasis (%)						
Yes	7	2 (29)	5 (71)	1	1 (100)	0 (0)
No	61	18 (30)	43 (70)	28	12 (43)	16 (57)
Liver metastasis (%)						
Yes	6	3 (50)	3 (50)	4	1 (25)	3 (75)
No	62	17 (27)	45 (73)	25	12 (48)	13 (52)
Bone metastasis (%)						
Yes	17	8 (47)	9 (53)	5	4 (80)	1 (20)
No	51	12 (24)	39 (76)	24	9 (38)	15 (62)
Elevated tumour markers (%)						
CA125	24	9 (38)	15 (62)	10	5 (50)	5 (50)
CEA	18	6 (33)	12 (67)	11	4 (36)	7 (64)
NSE	23	6 (26)	17 (74)	12	8 (67)	4 (33)
Cyfra21-1	44	15 (34)	29 (66)	20	10 (50)	10 (50)
ProGRP	7	2 (29)	5 (71)	3	2 (67)	1 (33)
SCC	30	11 (37)	19 (63)	14	8 (57)	6 (43)
Pathologic T stage (%)						
T1	3	1 (33)	2 (67)	2	1 (50)	1 (50)
T2	15	2 (13)	13 (87)	6	1 (17)	5 (83)
T3	14	5 (36)	9 (64)	11	4 (36)	7 (64)
T4	36	12 (33)	24 (67)	10	7 (70)	3 (30)
Pathologic N stage (%)						
N0	5	1 (20)	4 (80)	1	1 (100)	0 (0)
N1	6	3 (50)	3 (50)	3	1 (33)	2 (67)
N2	14	5 (36)	9 (64)	12	5 (42)	7 (58)
N3	43	11 (26)	32 (74)	13	6 (46)	7 (54)
Line of treatment (%)						
First line	35	2 (6)	33 (94)	13	2 (15)	11 (85)
Later line	33	18 (55)	15 (45)	16	11 (69)	5 (31)
Treatment strategy (%)						
Monotherapy	36	16 (44)	20 (56)	15	8 (53)	7 (47)
Combination therapy	32	4 (13)	28 (87)	14	5 (36)	9 (64)
PFS [M (Q_1_, Q_3_)]		3.3 (1.9, 4.7)	9.5 (6.5, 24.5)		2.8 (1.8, 14.4)	9.6 (7.3, 22.3)

Rapid and slow represent the RP and SP subgroups by delta radiomics scores. PFS, progression-free survival (months).

### Radiomics feature extraction and screening

**Figure 2:**
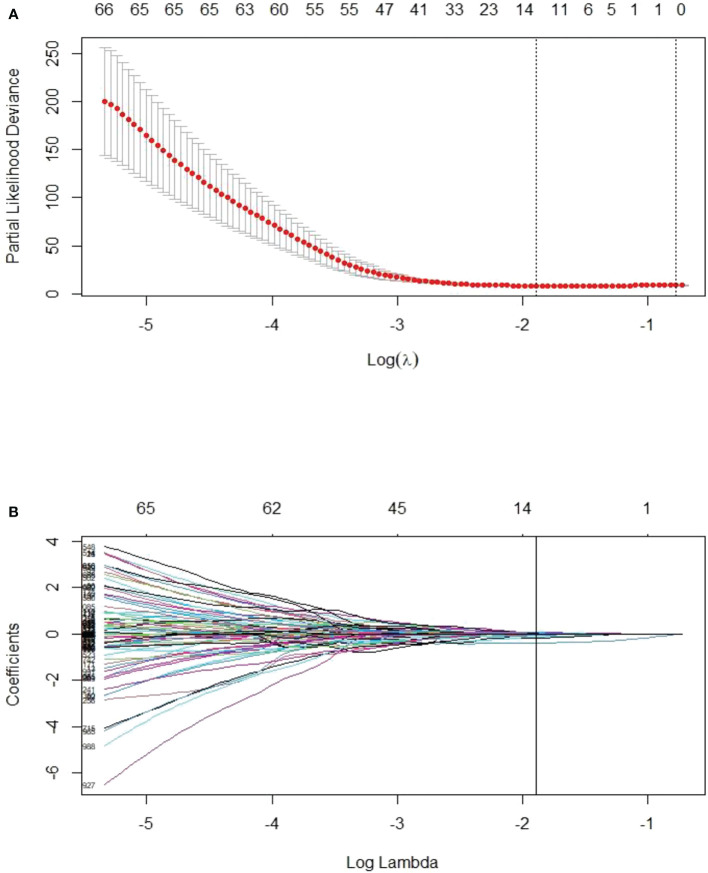
LASSO Cox regression model for delta radiomics feature screening. **(A)** The dashed line on the left side of the horizontal coordinate represents the selection of the best log(λ) = -1.8905 in the model by tenfold cross-validation. **(B)** Coefficient convergence plots of the screened features, with black vertical lines corresponding to the best log(λ) values, screened for 12 nonzero coefficients of delta RFs.

Among the four major categories (first order features, shape-based features, textural features, algorithmically transformed features) of the 1246 extracted RFs, the RFs with nonzero coefficients associated with PFS were screened according to the dimensionality reduction of LASSO Cox regression. Among them, three optimal features were obtained after screening TP0 RFs (**Supplementary Table S2**). Six optimal features were obtained after screening TP1 RFs (**Supplementary Table S3**). Twelve optimal features were obtained after screening delta RFs (**Figure 2**, **Supplementary Table S4**). The extracted RFs were all reproducible, with ICCs >0.85 (P < 0.05) for the two radiologists.

### Construction of the RM

The radiomics signatures were constructed according to the screened RFs and the corresponding weights (**Supplementary Equations 1, 2, 3**). Based on the radiomics signatures, the TP0 RM, TP1 RM, and delta RM were constructed. In the training cohort, the C-indexes of the three models were 0.64 (95%CI=0.57-0.71), 0.75 (95%CI=0.69-0.81), and 0.80 (95%CI=0.75-0.85), respectively. The delta RM had statistically significant differences compared with the TP0 RM and TP1 RM (P<0.0001). In the validation cohort, the C-indexes of the three models were 0.61 (95%CI=0.48-0.74), 0.68 (95%CI=0.54-0.82), and 0.78 (95%CI=0.68-0.88), respectively, and no obvious difference was found between the delta RM and the TP0 RM or the TP1 RM (P > 0.05).

### Delta rad-score stratification

**Figure 3:**
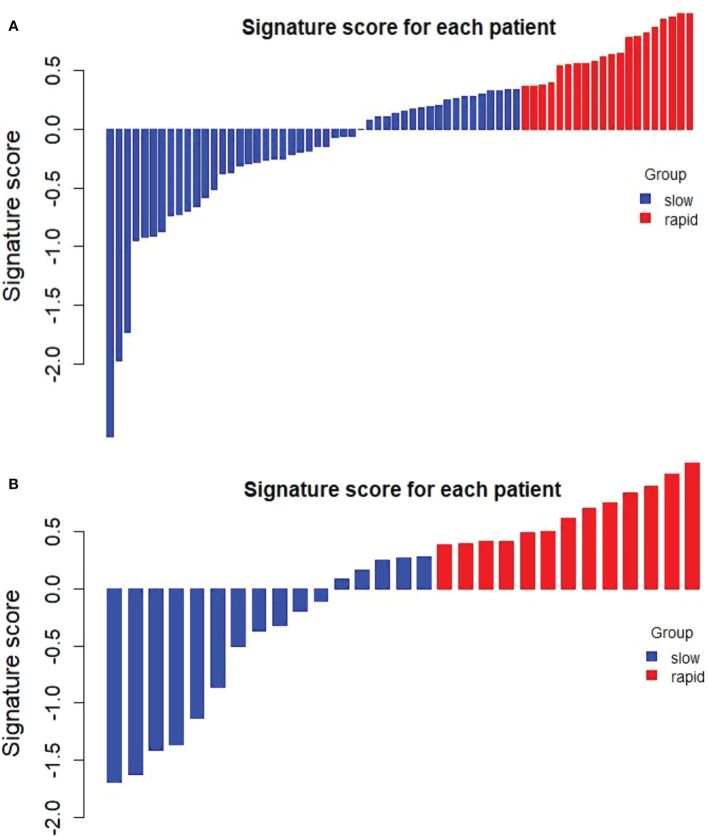
Distribution of delta radiomics scores in the training cohort **(A)** and validation cohort **(B)**. Delta radiomics scores above the cut-off value were categorized as the RP subgroup (red), and delta radiomics scores below the cut-off value were categorized as the SP subgroup (blue). There was an obvious difference in the distribution of the Delta Rad-score between the RP and SP subgroups.

**Figure 4:**
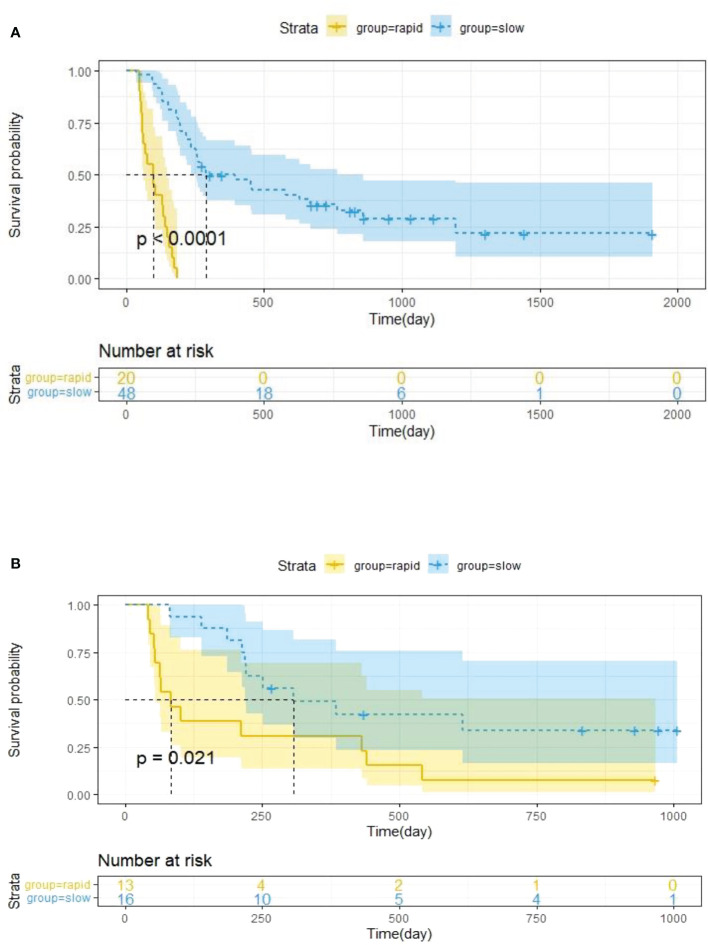
Kaplan–Meier survival analysis was performed in the training cohort **(A)** and validation cohort **(B)**, and PFS was markedly lower in the RP subgroup (yellow curve) than in the SP subgroup (blue curve). Statistical difference was assessed using the log-rank test.

The optimal cut-off value of the Delta Rad-score was determined to be 0.36 according to X-tile, and the patients were divided into a RP subgroup (Delta Rad-score ≥ 0.36) and a SP subgroup (Delta Rad-score < 0.36). An obvious difference was found in the distribution of the Delta Rad-score per patient in the training and validation cohorts (**Figure 3**). As expected with treatment with ICIs, there were more SP patients (blue bars) than RP patients (red bars) in the training and validation cohorts. The proportions of RP patients in the training and validation cohorts were 29% and 45%, respectively. The median PFS of the RP and SP subgroups in the training cohort were 3.3 (1.9-4.7) months and 9.5 (6.5-24.5) months, respectively. The median PFS of the RP and SP subgroups in the validation cohort were 2.8 (1.8 to 14.4) and 9.6 (7.3 to 22.3) months, respectively. The Kaplan–Meier survival curve proved the remarkable difference of PFS between the stratified RP and SP subgroups in the two cohorts, with a log-rank test of P<0.0001 and a hazard ratio (HR) of 10.233 in the training cohort and a log-rank test P < 0.05 and an HR of 2.633 in the validation cohort (**Figure 4**). This finding implies that this radiomics signature is effective in identifying patients at high risk of rapid progression.

### Nomogram construction and evaluation

**Figure 5:**
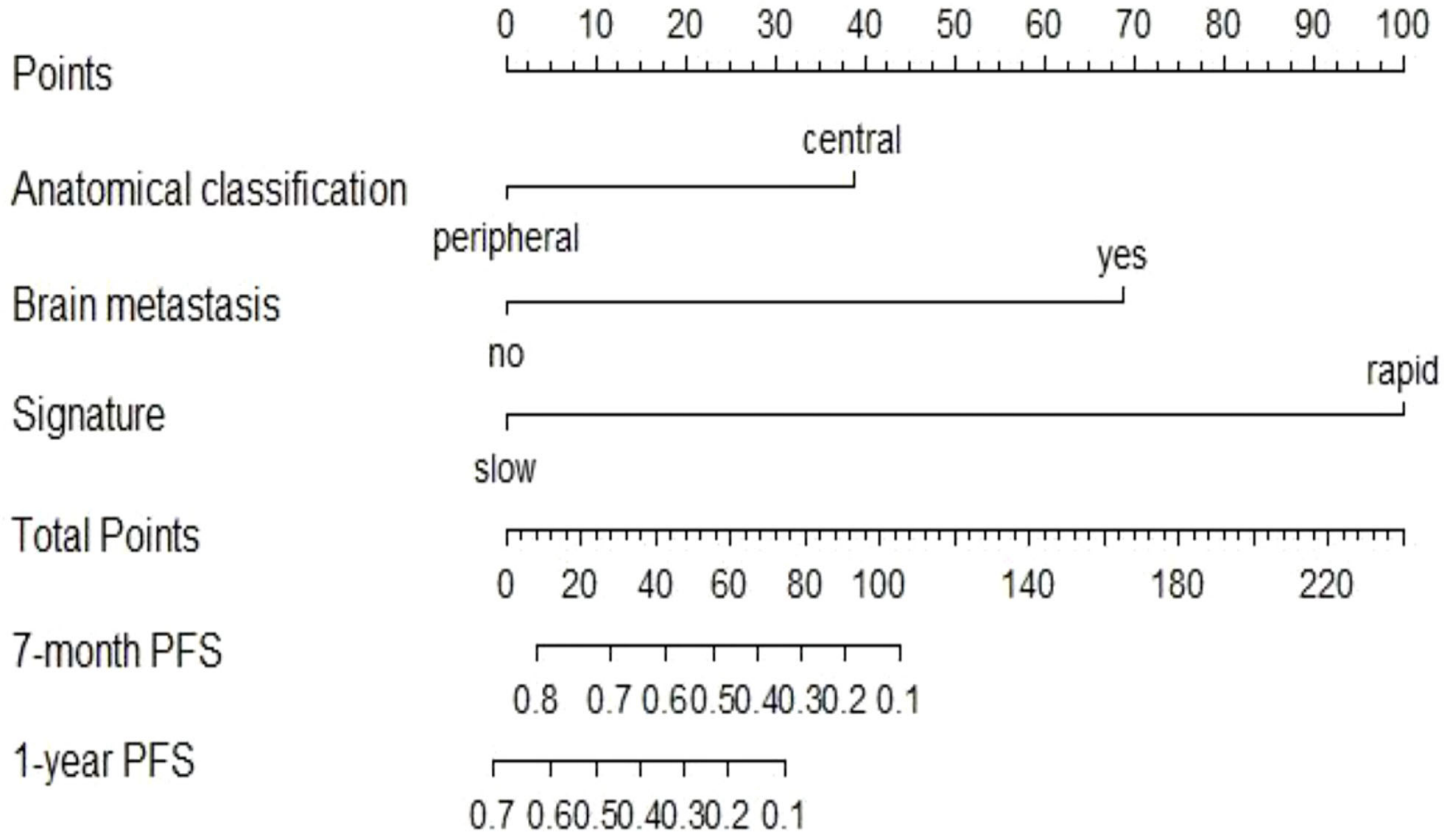
Nomogram to predict the 7-month and one-year PFS probabilities of NSCLC patients after ICI treatment.

The univariate Cox analysis showed that smoking history, tumour anatomical classification, brain metastasis, CA125, line of treatment, treatment strategy, and delta radiomics signature were associated factors affecting PFS in NSCLC patients treated with ICIs (P<0.05) (**Supplementary Table S5**). Multivariate Cox analysis demonstrated two clinicopathologic characteristics (tumour anatomical classification and brain metastasis) and the delta radiomics signature as independent predictors of PFS (P<0.05) (**Table 2**). A prediction model integrating these variables was developed and displayed as a nomogram (**Figure 5**). In the training cohort, Harrell’s C-indexes of the nomogram were 0.83 (95%CI=0.78-0.88) for the training cohort and 0.70 (95%CI=0.60-0.80) for the validation cohort. The C-indexes dropped to 0.66 (95%CI=0.59-0.73) and 0.62 (95%CI=0.53-0.72) in the training and validation cohorts, respectively, if the signature was excluded from the nomogram and retained only the significant clinicopathologic characteristics. There were statistically significant differences between the combined prediction model and the clinical prediction model (P<0.05) in the training and validation cohorts (**Supplementary Table S6**). The integration of the delta RFs into the nomogram significantly enhanced the prediction accuracy.

**Table 2:** Results of multifactorial Cox regression analysis of clinical variables and RFs in the training cohort.

Variables	Training cohort (N=68)	
	P value	HR (95%CI)
Anatomical classification (Central vs. Peripheral)	<0.01	2.515 (1.321, 4.786)
Brain metastasis (Yes vs. No)	<0.001	5.115 (2.060, 12.699)
Signature (Rapid progression vs. Slow progression)	<0.0001	10.982 (4.785, 25.206)

HR, hazard ratio; CI, confidence interval.

**Figure 6:**
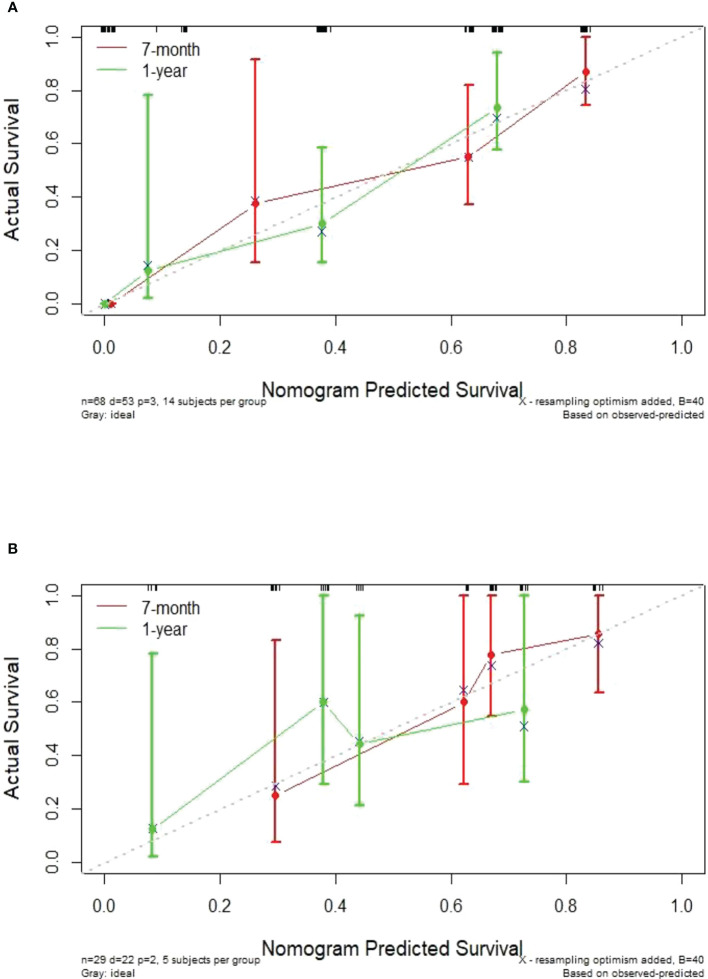
Calibration curves showing an excellent agreement between the predictions and observations of the 7-month and 1-year NSCLC progression probabilities in the training cohort **(A)** and validation cohort **(B)**.

**Figure 7:**
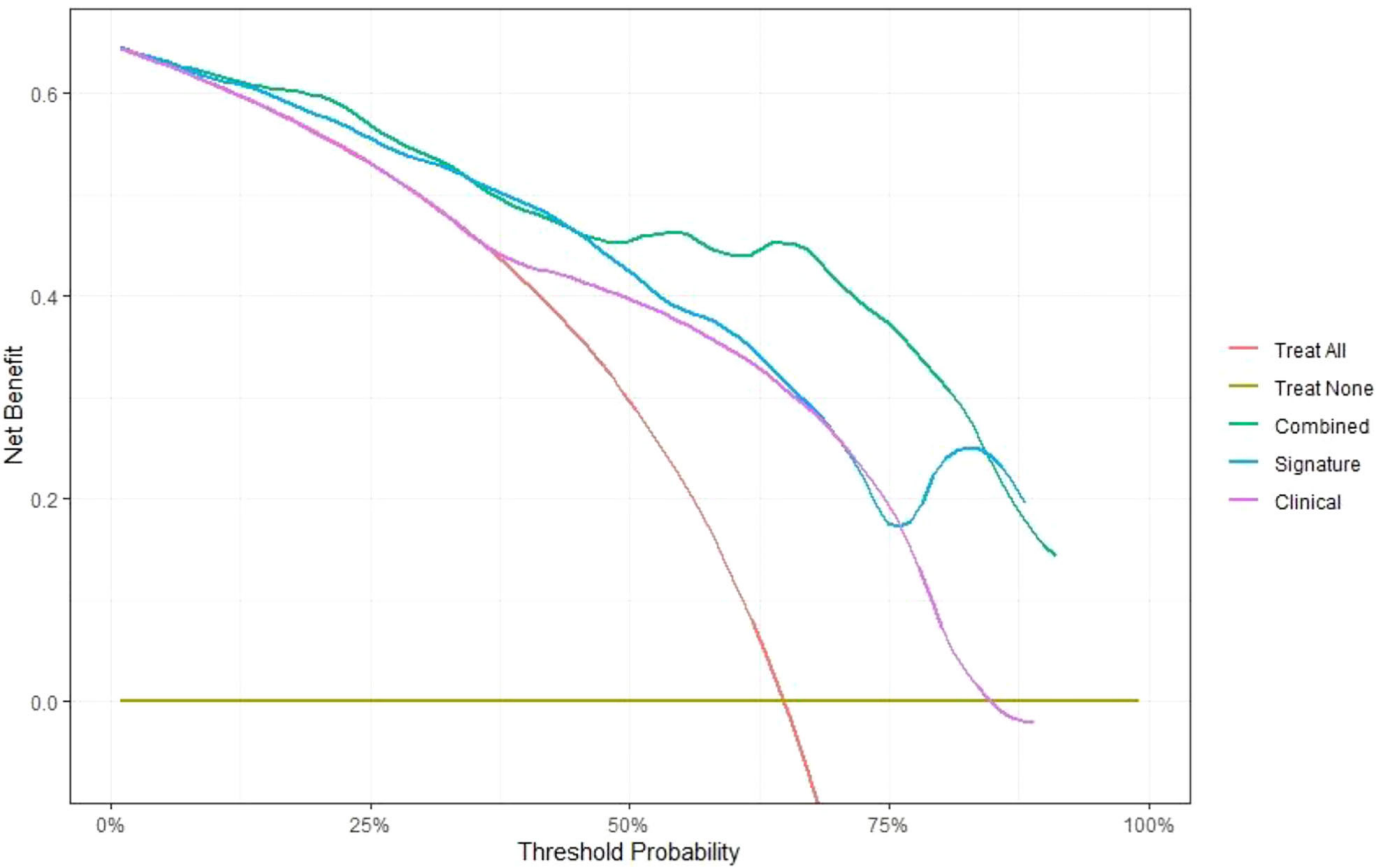
Comparison of the net benefits of the combined prediction model (green), delta radiomics prediction model (blue) and clinical prediction model (purple) using decision curve analysis.

In this study, the individualized nomogram calibration curves revealed good agreement between the prediction and observation results of the 7-month and 1-year NSCLC progression probabilities in the training and validation cohorts (**Figure 6**). DCA indicated that the combined prediction model could provide better clinical utility than the delta radiomics prediction model or the clinical prediction model within a reasonable threshold probability range (**Figure 7**).

### Comparison of the predictive efficacy of PD-L1 expression and radiomics

In our study cohort, the expression of PD-L1 was examined in 22 patients, which was positive in 12 of 22 patients (55%) and negative in the remaining patients. We used these 22 patients as an independent cohort to compare the Delta Rad-score and PD-L1 expression in predicting RP high-risk patients. The delta RM [C-index of 0.86 (95%CI=0.78-0.94)] had higher predictive accuracy than PD-L1 expression status [C-index of 0.50 (95%CI=0.36-0.64)], and the difference was significant (P<0.0001).

## Discussion

The survival of NSCLC patients varies greatly among individuals, and survival prognosis is influenced by multiple factors. We used a radiomics approach to analyse the CT feature-based signature for monitoring the treatment efficacy of ICIs in advanced NSCLC patients. Most previous studies (38–40) constructed single time-point RMs from baseline CT scans for predicting patient outcomes, but they did not contain information about treatment response. Delta radiomics can provide massive data on treatment-induced changes, contains rich time-dependent information, dynamically assesses tumour burden and is more consistent with the assessment of immunotherapy efficacy in clinical practice. We used the LASSO Cox regression model to screen critical features with a significant correlation with PFS based on pretreatment, posttreatment and delta features, developed and compared pretreatment, posttreatment and delta RMs. The delta RFs were identified and compared to the single-time-point RFs at TP0 and TP1. Interestingly, the delta RM exhibited a higher C-index than the TP0 and TP1 RMs in both the training and validation cohorts, which agrees with recent literatures (41, 42). The lower 95%CIs of the C-index (0.48 for TP0, 0.54 for TP1) indicated insufficient diagnostic efficiency of TP0 and TP1 in the validation cohort, although the C-index did not differ in the TP0, TP1 and delta RM. We speculate that this is associated with the small sample size of our study and that a larger patient population is still required for validation. Our results show that the delta RM could provide better predictive accuracy and was the best model of the three RMs for predicting PFS in NSCLC. Compared with a recent similar study, Zerunian et al. (43) extracted texture features on baseline scan images to predict PFS of IV stage NSCLC treated with an anti-PD-1 therapy, with an AUC of 0.74, while in our study, the maximum predictive accuracy of delta RM was 0.86 (C-index), which is encouraging. Thus, it can be inferred that delta radiomics can give a better predictive decision support. The delta radiomics signature was used to assess the likelihood of patient survival risk stratification. Finally, we successfully stratified the patients into RP and SP subgroups using Kaplan–Meier survival curve analysis. For these rapidly progressing patients, their treatment strategies and follow-up need to be more thorough and careful.

The multivariable Cox analysis indicated two clinicopathologic characteristics (tumour anatomical classification and brain metastasis) and the signature as significant risk factors for predicting PFS in patients treated with ICIs. The location of the lung tumour is an important factor affecting treatment and survival, but research focused on the prognostic values of primary tumour location (central and peripheral types) in lung cancer is still in lack (44). Our study identified tumour anatomical classification as a significant risk factor for predicting PFS in NSCLC patients treated with ICIs by multifactorial Cox analysis. Central lung cancer has a higher rate of lymphatic metastasis and higher cytologic and histologic grades than peripheral lung cancer (44, 45), which are hypothesized to be related to the poor outcome of ICIs for central lung cancer. In this study, our analysis of patients with central lung cancer revealed that four (8%) patients with central lung cancer with hilum macrovascular erosion were all in the RP group. We also observed that 20 patients (38%) with central lung cancer had lobe or unilateral complete atelectasis at TP0, and only 10 patients (all in the SP group) showed significant improvement at TP1. In addition, we found 6 patients (11%) with mild or no obstructive pulmonary lesions at TP0, which worsened to whole lobe atelectasis at TP1 (all in the RP group). Another independent risk factor was brain metastasis status. Brain metastases occur at a high rate in NSCLC and are usually as a poor prognostic feature (46, 47). Remon et al. (48) and Sun et al. (49) both reported OS times of 3-14.8 months and 3-6 months, respectively, for NSCLC patients with brain metastases. Due to the lack of effective treatments, patients have decreased life quality and poor prognosis. To further clarify any additional benefits we could gain for predicting individualized PFS by combining radiomics signatures, we developed and compared predictive nomograms with and without radiomics signatures. We found that the combined prediction model was remarkably better than the clinical prediction model in predicting the individual prognosis of NSCLC patients in all cohorts. Thus, in this study, the combined prediction model including clinicopathologic characteristics (tumour anatomical classification and brain metastasis) and the delta radiomics signature could provide a better ability to predict prognosis.

Additionally, we compared the RM and PD-L1 expression status in terms of their predictive ability for RP high-risk patients. The results demonstrated that the delta RM was superior to PD-L1 expression in predicting the therapeutic response of ICIs in NSCLC patients, consistent with the results reported in the literature (40). Researchers could further validate the PD-L1 expression characteristics combined with the delta RM in prospective trials to better stratify patients for outcome prediction.

Our study has some limitations. First, in this work, we selected the largest primary lung tumour to extract features without including all target lesions (2 lesions per organ and 5 lesions per patient) for analysis, which to some extent failed to reflect the total tumour load. Second, the sample size of the cohort was quite small, and the robustness and validity of the model need to be validated using a larger dataset. Third, the underlying biological basis of the radiological features is not discussed in the current study, as the 12 key features extracted already cover four major categories of radiomics. Fourth, patients who received both anti-PD-1 or anti-PD-L1 monotherapy and immunotherapy-based combination therapy in our cohort were included in the dataset, which led to heterogeneity in our cohort. By stratifying the treatment modalities, although univariate analysis showed an association with efficacy, the final results confirmed that it was not a significant risk factor and that no obvious difference in efficacy was observed between treatment strategy groups. Further prospective study with larger datasets is warranted to ensure the predictive efficacy of CT-based RMs in predicting ICI resistance.

In summary, we developed a noninvasive delta RM based on CT imaging to stratify the survival risk of stage III-IV NSCLC patients treated with ICIs. We then combined this feature with clinicopathological features (tumour anatomical classification and brain metastasis status) to construct a nomogram for PFS prediction, which could help to clinically guide the development of individualized treatment plans and increase the chances of survival of these patients.

## Data availability statement

The original contributions presented in the study are included in the article/**Supplementary Material**. Further inquiries can be directed to the corresponding author.

## Ethics statement

The study was approved by the hospital ethics committee (Grant No.: Research 20220222-33), and was carried out in compliance with the Declaration of Helsinki.

## Author contributions

DX: conceptualization, methodology, software, data curation, visualization, writing - original draft, writing - review and editing. FX: conceptualization, methodology, formal analysis, writing - review and editing. WZ: software, data curation, visualization, writing - review and editing. CP: conceptualization, methodology, investigation, writing - review and editing. SH: data curation, visualization, writing - review and editing. KL and DH: validation, writing - review and editing. YW: investigation, writing - review and editing. CH: data curation, writing - review & editing. HH: conceptualization, resources, project administration, funding acquisition. All authors contributed to the article and approved the submitted version.

## Funding

This research was supported by Zhejiang Provincial Natural Science Foundation of China under grant No. LQ20F030018, National Natural Science Foundation of China under grant 82071988, Key Research and Development Program of Zhejiang Province under grant 2019C03064, Program Co-sponsored by Province and Ministry under grant WKJ-ZJ-1926.

## Conflict of interest

The authors declare that the research was conducted in the absence of any commercial or financial relationships that could be construed as a potential conflict of interest.

## Publisher’s note

All claims expressed in this article are solely those of the authors and do not necessarily represent those of their affiliated organizations, or those of the publisher, the editors and the reviewers. Any product that may be evaluated in this article, or claim that may be made by its manufacturer, is not guaranteed or endorsed by the publisher.
