# Supplementary material for: Delta radiomics model for the prediction of progression-free survival time in advanced non-small-cell lung cancer patients after immunotherapy
Source: Front Oncol. 2022 Oct 6;12:990608. doi: 10.3389/fonc.2022.990608 (PMC9583844; doi:10.3389/fonc.2022.990608)
Supplement: Supplementary file 1 [file DataSheet_1.docx]

Supplementary Material

# Supplementary Data

**CT scanning protocol**

The scanning parameters were as follows: tube voltage = 120 kV, tube current = 200-300 mA, rotation time = 0.75 s, detector collimation = 32x1.25 mm, field of view (FOV) = 36.00-50.00 cm, and pixel matrix 512x512. After routine nonenhanced CT, contrast-enhanced CT was performed after a 25-30 s delay following intravenous administration of 1.1-1.7 ml/kg iodinated contrast material at a rate of 2.5–3.0 mL/s with a pump injector. CT images were reconstructed using a slice thickness of 1.25 mm with the standard kernel.

**Supplementary Equations**

Supplementary Equation 1:

TP0 radiomics signature = [-0.0102 × feature (1)] + [-0.0803 × feature (2)] + 0.0611× feature (3). Supplementary Equation 2:

TP1 radiomics signature = 0.0310 × feature (1) + 0.0694 × feature (2) + 0.0982 × feature (3) + [-0.0283 × feature (4)] + [-0.0773 × feature (5)] + [-0.1961 × feature (6)].

Supplementary Equation 3:

Delta radiomics signature = [-0.3918 × feature (1)] +0.0399 × feature (2) +0.0147 × feature (3) + [-0.1816 × feature (4)] + 0.0779 × feature (5) + 0.0266 × feature (6) + [-0.0547 × feature (7)] + 0.0075 × feature (8) + [-0.1269 × feature (9)] + [-0.0525 × feature (10)] + 0.0517 × feature (11) + 0.0604 × feature (12).

# Supplementary Tables

**Table S1.** Demographic and clinicopathologic characteristics and PFS of the training and validation cohorts. PFS: progression-free survival (months); ^#^Mann-Whitney test; *Fisher's exact test.

| Demographic or clinicopathologic  characteristic, PFS | Training cohort（N=68） | Validation cohort（N=29） | P value |
| --- | --- | --- | --- |
| Sex (%) |  |  | 1.000 |
| Male | 62(91) | 26(90) |  |
| Female | 6(9) | 3(10) |  |
| Age, years (%)  ≤65  ＞65 | 25(37)  43(63) | 16(55)  13(45) | 0.093 |
| Smoking history (%) |  |  | 0.982 |
| Smoker | 35(51) | 15(52) |  |
| Non-smoker | 33(49) | 14(48) |  |
| Anatomical classification (%) |  |  | 0.607 |
| Central type | 36(53) | 17(59) |  |
| Peripheral type | 32(47) | 12(41) |  |
| Pathological type (%) |  |  | 0.196 |
| Squamous cell | 45(66) | 23(79) |  |
| Adenocarcinoma | 23(34) | 6(21) |  |
| Lung metastasis (%) |  |  | 0.142 |
| Yes | 20(29) | 13(45) |  |
| No | 48(71) | 16(55) |  |
| Brain metastasis (%) |  |  | 0.272 |
| Yes | 8(12) | 1(3) |  |
| No | 60(88) | 28(97) |  |
| Liver metastasis (%) |  |  | 1.000 |
| Yes | 6(9) | 3(10) |  |
| No | 62(91) | 26(90) |  |
| Bone metastasis (%) |  |  | 0.404 |
| Yes | 17(25) | 5(17) |  |
| No | 51(75) | 24(83) |  |
| Elevated tumour markers (%) |  |  |  |
| CA125 | 24(35) | 10(34) | 0.139 |
| CEA | 18(26) | 11(38) | 0.259 |
| NSE | 23(34) | 12(41) | 0.478 |
| Cyfra21-1 | 44(65) | 20(69) | 0.685 |
| ProGRP | 7(10) | 3(10) | 1.000 |
| SCC | 30(44) | 14(48) | 0.171 |
| Pathologic T stage (%) |  |  | 0.215* |
| T1 | 3(4) | 2(7) |  |
| T2 | 15(22) | 6(21) |  |
| T3 | 14(21) | 11(38) |  |
| T4 | 36(53) | 10(34) |  |
| Pathologic N stage (%) |  |  | 0.177* |
| N0 | 5(7) | 1(3) |  |
| N1 | 6(9) | 3(10) |  |
| N2 | 14(21) | 12(41) |  |
| N3 | 43(63) | 13(45) |  |
| Line of treatment (%) |  |  | 0.549 |
| First line | 35(51) | 13(45) |  |
| Later line | 33(49) | 16(55) |  |
| Treatment strategy (%) |  |  | 0.912 |
| Monotherapy | 36(53) | 15(52) |  |
| Combination therapy | 32(47) | 14(48) |  |
| PFS [M (Q1, Q3)] | 6.9(4.3,19.7) | 7.3(2.8,14.7) | 0.724^#^ |

**Table S2.** Three screened TP0 radiomics features and their weights.

|  | Feature Name | Grouping | Transformation | Weights |
| --- | --- | --- | --- | --- |
| R1 | InverseVariance | GLCM | log-sigma-4-0-mm-3D | -0.0102 |
| R2 | DependenceEntropy | GLDM | wavelet-HLL | -0.0803 |
| R3 | MCC | GLCM | wavelet-LLL | 0.0611 |

**Table S3.** Six screened TP1 radiomics features and their weights.

|  | Feature Name | Grouping | Transformation | Weights |
| --- | --- | --- | --- | --- |
| R1 | Maximum | Firstorder | None | 0.0310 |
| R2 | LongRunHighGrayLevelEmphasis | GLRLM | log-sigma-2-0-mm-3D | 0.0694 |
| R3 | LargeDependenceEmphasis | GLDM | log-sigma-3-0-mm-3D | 0.0982 |
| R4 | Skewness | Firstorder | log-sigma-5-0-mm-3D | -0.0283 |
| R5 | InverseVariance | GLCM | log-sigma-5-0-mm-3D | -0.0773 |
| R6 | Imc2 | GLCM | wavelet-HLL | -0.1961 |

**Table S4.** Twelve screened delta radiomics features and their weights.

|  | Feature Name | Grouping | Transformation | Weights |
| --- | --- | --- | --- | --- |
| R1 | SurfaceVolumeRatio | Shape | None | -0.3918 |
| R2 | 90Percentile | Firstorder | None | 0.0399 |
| R3 | Maximum | Firstorder | None | 0.0147 |
| R4 | ShortRunLowGrayLevelEmphasis | GLRLM | log-sigma-2-0-mm-3D | -0.1816 |
| R5 | DependenceVariance | GLDM | log-sigma-2-0-mm-3D | 0.0779 |
| R6 | LargeAreaHighGrayLevelEmphasis | GLSZM | log-sigma-4-0-mm-3D | 0.0266 |
| R7 | firstorder_Skewness | Firstorder | wavelet-LHH | -0.0547 |
| R8 | DependenceVariance | GLDM | wavelet-LLH | 0.0075 |
| R9 | ShortRunLowGrayLevelEmphasis | GLRM | wavelet-LHL | -0.1269 |
| R10 | SmallDependenceLowGrayLevelEmphasis | GLDM | wavelet-LHH | -0.0525 |
| R11 | Idn | GLCM | wavelet-HLH | 0.0517 |
| R12 | SizeZoneNonUniformity | GLSZM | wavelet-HHH | 0.0604 |

**Table S5.** Results of univariate Cox regression analysis of clinical variables and radiomics features in the training cohort. HR: hazard ratio; CI: confidence interval.

| Variables | Training cohort (N=68) | |
| --- | --- | --- |
|  | P value | HR (95% CI) |
| Sex(Male vs. Female) | 0.660 | 0.812（0.321，2.051） |
| Age, years (>65 vs. ≤65) | 0.230 | 1.425（0.799，2.542） |
| Smoking history (Smoker vs. Non-smoker) | 0.030 | 1.834（1.060，3.174） |
| Anatomical classification (Central vs. Peripheral) | 0.002 | 2.446（1.398，4.279） |
| Pathological type (Squamous cell vs. Adenocarcinoma) | 0.640 | 1.151（0.639，2.072） |
| Lung metastasis (Yes vs. No) | 0.373 | 1.307（0.725，2.358） |
| Brain metastasis (Yes vs. No) | 0.036 | 2.359（1.055，5.271） |
| Liver metastasis (Yes vs. No) | 0.390 | 1.500（0.595，3.785） |
| Bone metastasis (Yes vs. No) | 0.400 | 1.302（0.704，2.409） |
| CA125 (Elevated vs. Normal) | 0.036 | 1.812（1.041，3.154） |
| CEA (Elevated vs. Normal) | 0.176 | 1.513（0.830，2.760） |
| NSE (Elevated vs. Normal) | 0.692 | 1.119（0.641，1.954） |
| Cyfra21-1 (Elevated vs. Normal) | 0.851 | 1.055（0.601，1.852） |
| ProGRP (Elevated vs. Normal) | 0.883 | 0.937（0.396，2.221） |
| SCC (Elevated vs. Normal) | 0.080 | 1.635（0.943，2.836） |
| T stage T1 as reference: T2 | 0.478 | 0.393（0.106，1.459） |
| T3 | 0.175 | 0.556（0.152，2.039） |
| T4 | 0.675 | 0.648（0.196，2.145） |
| N stage N0 as reference: N1 | 0.189 | 4.874（0.970，24.487） |
| N2 | 0.165 | 3.922（0.879，17.498） |
| N3 | 0.219 | 2.613（0.623，10.948） |
| M stage (M0 vs. M1) | 0.829 | 1.062（0.616，1.829） |
| Line of treatment (First line vs. Later line) | 0.006 | 0.461（0.266，0.797） |
| Treatment strategy (Monotherapy vs. Combination therapy) | 0.045 | 1.757（1.012，3.050） |
| Signature (Rapid progression vs. Slow progression) | ＜0.0001 | 10.233（4.502，23.258） |

**Table S6.** Comparison of training and validation prediction models. HR: hazard ratio; CI: confidence interval.

| Models | Training cohort | | | Validation cohort | | |
| --- | --- | --- | --- | --- | --- | --- |
|  | C-index | 95%CI | P value | C-index | 95%CI | P value |
| Combined prediction model | 0.83 | 0.78～0.88 |  | 0.70 | 0.60～0.80 |  |
| Clinical prediction model | 0.66 | 0.59～0.73 | ＜0.0001 | 0.62 | 0.53～0.72 | ＜0.05 |
| Delta radiomics prediction model | 0.79 | 0.73～0.85 | ＜0.0001 | 0.65 | 0.55～0.75 | ＞0.05 |
